# Genetically modified (GM) late blight-resistant potato and consumer attitudes before and after a field visit

**DOI:** 10.1080/21645698.2022.2133396

**Published:** 2022-10-20

**Authors:** Jéssica Bubolz, Patrycja Sleboda, Anna Lehrman, Sven-Ove Hansson, Carl Johan Lagerkvist, Björn Andersson, Marit Lenman, Svante Resjö, Marc Ghislain, Muhammad Awais Zahid, Nam Phuong Kieu, Erik Andreasson

**Affiliations:** a Department of Plant Protection Biology, Swedish University of Agricultural Sciences, Alnarp Campus, Sweden; b Deparment of Economics, Swedish University of Agricultural Sciences, Uppsala, Sweden; c International Potato Center (CIP), Nairobi, Kenya; d Department of crop prodction ecology, Swedish University of Agricultural Sciences, Uppsala, Swedan; e Department of forest mycology and plant pathology, Swedish University of Agricultural Sciences; f Department of Plant Protection Biology, Swedish University of Agricultural Sciences, Alnarp

**Keywords:** GM foods, GM potato, IPM, late blight, phytophthora infestans, R-genes

## Abstract

Late blight, caused by *Phytophthora infestans*, is the most devastating disease in potato production. Here, we show full late blight resistance in a location with a genetically diverse pathogen population with the use of GM potato stacked with three resistance (R) genes over three seasons. In addition, using this field trials, we demonstrate that in-the-field intervention among consumers led to change for more favorable attitude generally toward GM crops.

## Introduction

Potato (*Solanum tuberosum* L.) is the world’s third most important crop for human consumption.^1^ Potato late blight, caused by the oomycete *Phytophthora infestans*, reportedly causes annual losses of 16% of total potato production,^2^ with an estimated annual global cost of €6.1 billion. Chemical control methods make important contributions to crop protection, but they are costly and a recent European Union (EU) resolution included a goal to reduce use of pesticides by 50% by 2030.^3^ This calls for implementation of novel sustainable approaches to control late blight, such as stacking of genetic resistance by cis/trans genesis or gene editing, especially since conventional introgression of several R genes from wild relatives may take several decades in potato breeding due to linkage drag of undesired genes.^4^ No major market potato cultivars with stacked R genes are yet available in Europe.

Novel food production methods will be essential agricultural elements of a sustainable future,^5^ and crucial to meet urgent needs for adaptation to anticipated climate change.^6^ However, despite scientists widely agreeing that crop cultivars developed using gene technology in plant breeding are as safe for human consumption as conventionally bred cultivars,^7,8^ Extensive research shows that genetically modified (GM) food evokes strong, mostly negative attitudes among consumers. ^9–14^ As attitudes in general correspond with consumers’ preferences, acceptance, and willingness to purchase^15^ similar findings were observed in the GM food context,^16–20^ misperceptions about the risks and benefits should be addressed with communication that focuses on closing gaps in knowledge regarding novel food technologies and engaging citizens in debate. Indeed, individuals with greater knowledge about GM food has been found be holding a more positive attitude^21,22^ and to be more likely to accept GM foods,^23,24^ while rejection of GM products was found to be associated with limited knowledge about GM.^25^ However, it may not be effective to only provide fact-based information while ignoring the risk of motivated reasoning triggered by existing beliefs or knowledge.^26^ Previous research has tested the effectiveness of communication for attitude change, with either messages about benefits or risks of GM foods, and found either no change in attitudes^9,27^ or an unintended change toward more negative attitudes.^28–31^ However, providing fact-based information while ignoring the risk of triggering contrary reasoning rooted in existing beliefs or knowledge^26^ is not successful, leading either to no change^28,29^ or to an opposite to intended – shifts toward more negative attitudes.^28–31^ Only few studies have been found succesfull in changing attitudes toward GM food for more favorable.^20, 32–34^Thus, policymakers, scientists and communication specialists have to find ways to address and engage with consumers’ concerns regarding use of novel technologies in food production.

Our over-arching aims were 1) to evaluate the resistance of 3R stacked GM lines to local complex late blight population under field conditions; and 2) investigate the impact of in-the-field-intervention and personal experience with GM potato on consumers’ attitudes, risk perception toward to gene technology in plant breeding and willingness to purchase GM product. Furthermore, we investigated the effect of the R-genes in tubers, considering that different R-genes may provide different levels of tuber blight resistance.^35–37^

## Material and Methods

### Plant Material

The transgenic potatoes used in the experiment were obtained from the cultivar King Edward, the most common cultivar in Sweden.^38^ King Edward plants stacked with three R-genes (*RB* and *Rpi-blb2* from *Solanum bulbocastanum*, and *Rpi-vnt1.1* from *Solanum venturi*) were produced using *Agrobacterium tumefaciens* following published procedures.^39^ Three transformed lines were selected for testing in this study (designated KE_3R_4, KE_3R_14, and KE_3R_43) in addition to the non-transformed King Edward line, all kept under the same in vitro conditions.^39^ Tubers for seeding were multiplied in a greenhouse for the first season, again following published procedures.^40^

### Detached Leaf Assay

*Phytophthora infestans* strain 88069 was cultivated on solid rye sucrose medium,^41^ in Petri dishes incubated at 18°C in darkness, and sub-cultured every three to four weeks. Sporangia were harvested by flooding 14-day-old cultures with cold (4°C) deionized water and gentle rubbing. The resulting suspensions were filtered through 40 μm nylon mesh and concentrated to 50 000 sporangia/mL. Twenty-five µL of the spore solution was pipetted and the leaves were maintained in a humid environment (RH ~ 100%) under controlled conditions.^42^ Results were recorded by measuring the infection size of each leaflet at 7 days post inoculation (dpi).

### Tuber Blight Assay

Tubers were inoculated with a suspension of 15 000 sporangia/mL of *P. infestans* strain 88069. In total, 40 whole tubers were used: 10 of each line (three 3 R lines and control, King Edward, lines). The whole tubers were washed and halved,^43^ then the halves were randomly distributed in plastic boxes and each was inoculated with 20 µL of the inoculum suspension. After inoculation, they were kept at 16–18°C in darkness for 12 days, and 100% relative humidity for at least the first 24 hours.

### Field Trials

Field trials of spontaneous infection by *P. infestans* were carried out in three consecutive years (2019, 2020, and 2021) at an established field trial site with at least four-year crop rotations in southern Sweden (Borgeby, geographic position 55.75289, 13.04872) using a randomized block design with four replicates. Each of the replicate consisted of a row of ten plants and the experiment was only sprayed with mineral oil.^44^ The entire plot was surrounded by a row of untreated potato cultivar Bintje. A permit for the field-trials was granted by the Swedish Board of Agriculture (Dnr 4.6.18–10775/16). The trials were conducted in accordance with the requirements exposed in the ‘Environmental Code’ (1998;808), the Code of Regulations of the Swedish Board of Agriculture (SJVFS 2003:5) on transport and labeling, as well as Regulation 2002:1086 on deliberate release of GMOs to the environment.

### Late Blight Field Scoring

The severity of late blight symptoms was visually scored twice a week toward the end of the growing season, from early July to late August, as previously described. The assigned scores ranged from 0 (no observed disease) to 100% (plant completely dead with no green leaves).

*P. infestans* genotyping

FTA-cards were used for pathogen sampling.^45^ In each test, a leaflet with a single lesion was pressed with the sporulating side facing down on the sampling area of the FTA and plant residues were removed. The FTA-cards were dried and stored at room temperature until they were packed and sent to the James Hutton Institute for genotyping by DNA fingerprinting using a 12-plex Simple Sequence Repeats (SSRs) method.^46^ The genotype data were analyzed using the Minimum Spanning Network clustering approach.^47^

### Consumer Attitude Questionnaire

Methods: Through social media channels of the Swedish University of Agricultural Sciences, a visit to a potato field trial was advertised. The information stated that the participants would meet researchers and get to know more about how potatoes could get more environmentally friendly and tastier, and that they could contribute to science by participation in a survey. No information about gene technology was included. Twenty-eight (28) Swedish citizens (14 female, 53% older than 50 years, 33% between 30 and 50 years old and 5% younger than 30 years) volunteered to join a trip to the field site of the study. Upon arrival no information about the technology was given. On the way to the field, they were asked to participate, voluntarily, in a paper-and-pencil survey and all subjects were informed that they could withdraw at any time. Those who participated received an envelope with a randomly assigned number. Each envelope included two smaller envelopes, one with a baseline questionnaire that they were asked to complete on the way to the field, and the other with a post-intervention questionnaire they were asked to fill on the way back from the field, after a 2- to 3-hour visit. Both questionnaires included the same questions.

Materials: Attitudes and risk perception were assessed with 9-items questionnaire followed by willingness to purchase (see Table 1). Participants were asked to respond using a Likert-point scale from 1 (strongly disagree) to 7 (strongly agree). The questions were adapted from prior studies on behavioral science and risk perception previously validated and tested among Swedish representative sample.^20^

**Table 1.** Questionnaire items for attitude, risk, and willingness to buy measures.

	Baseline	Post-intervention
	Mean (SD)	Mean (SD)
Plant breeding is not very important since we have land races and other good crop varieties that we can use in the future as well	1.96 (1.45)	2.46 (1.29)
Using genetic modification in plant breeding leads to unacceptable risks	4.04 (1.95)	3.64 (2.00)
Plant breeders using gene technology such as GM are helping big business more than they are helping farmers and consumers	4.82 (1.79)	4.33 (1.73)
If we use genetic modification in the right way, it can provide us with crops that are healthier and better for the environment	5.25 (1.71)	5.25 (1.69)
I generally perceive the application of gene technology in plant breeding as risky	4.61 (1.93)	3.62 (2.12)
I am sure I would never buy GM foodstuffs	2.96 (1.93)	2.54 (2.08)
I would buy genetically modified potato if it were more healthy, and the price is the same as for other potatoes.	4.79 (2.11)	5.12 (2.03)
I would buy genetically modified potato if it were 5 Swedish crowna cheaper per kilo than the other potatoes.	3.07 (2.32)	3.60 (2.52)
I would buy genetically modified potato if it were also organic	4.61(2.04)	5.00 (2.10)

All items were evaluated on a scale from 1 (strongly disagree) to 7 (strongly agree).

## Results and Discussion

**Figure 1.**
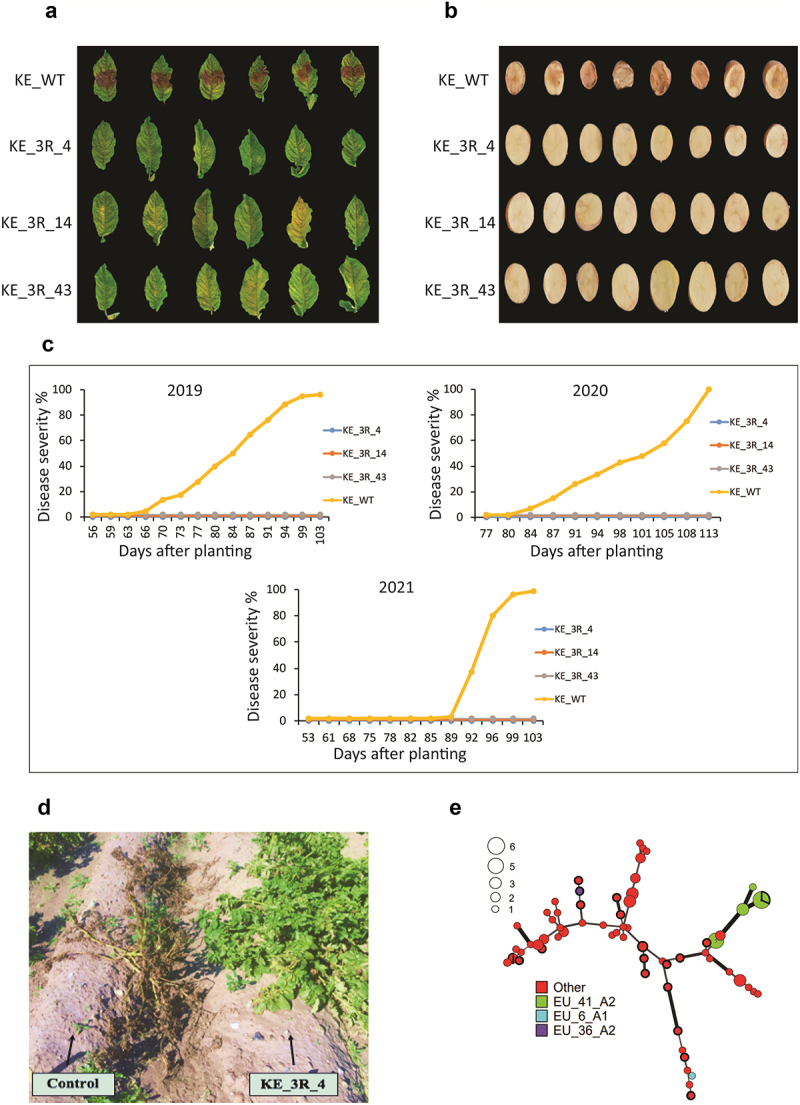
Detach leaflet analysis of inoculation with Phytophthora infestans 88069 of leaves (a) and tubers (b) under controlled conditions. C. Late blight (caused by *Phytophthora infestans*) scoring in untreated field experiments from three years in Sweden (2019–2021). D Photograph of KE_WT and KE_3 R_4 plants in 2020. E. Minimum-spanning network obtained from analysis of evolutionary relationships of *Phytophthora infestans* genotypes detected in samples collected in southern Sweden during 2019–2021. Circles with bold circumferences indicate isolates of *P. infestans* sampled at the site of the field trials, and empty circles show numbers of isolates represented by each circle in the network.

The goals of this investigation was to evaluate the late blight resistance of transgenic potatoes consisting of three *R* genes (*RB, Rpi-blb2*, and *Rpi-vnt1.1*) stacked in a dominant local potato cultivar under complex pathogen population, and use the trial for an in-field intervention with consumers. Late blight resistance genes may have varying degrees of efficacy, depending on diverse factors including the tissue, so we evaluated both leaf and tuber resistance. As expected from literature, no visual symptoms of disease were seen on 3 R potato leaves following inoculation with the 88069 isolate in a controlled environment (Fig. 1a). Analyses of tubers revealed that the 3 R potato lines also exhibited full resistance against tuber blight (Fig. 1b). Moreover, no evidence of disease with natural infections of *P. infestans* was detected in the 3 R potato lines during three consecutive years of field trials, while the non-transgenic (King Edward) variety was severely affected (Fig. 1c-d).

Field evaluations of late blight resistance are important in Swedish conditions, partly due to high genetic variation in the local *P. infestans* populations and presence of some highly virulent clonal lineages (https://agro.au.dk/forskning/internationale-platforme/euroblight/pathogen-monitoring/genotype-map). In genotyping of *P. infestans* samples from the 3 R fields and other fields in Southern Sweden during the three years the most frequently detected lineage was EU_41_A2, the EU_36_A2 genotype was found three times, but most detected genotypes were only found in one sample and classified as “other” (Fig. 1e). EU_41_A2 is a highly virulent clonal lineage^48^ that has established in Sweden since 2016. Our results reveal similar field resistance to the reported resistance arising from stacking the same three R-genes in potato cultivars Desiree and Victoria in an African highland region with a mainly clonal 2_A2 of *P. infestans*.^49^ We also found that the 3 R tubers could not be infected by *P. infestans* even after more than one year of storage period. This finding is important because, unlike existing late blight susceptible or partially resistant varieties, it prevents the spread of *P. infestans* strains through seed tubers within countries and even between continents.^50^ In addition, in countries where tubers are stored for later consumption, it contributes to food security by minimizing storage losses due to diseased (rotten) tubers.

**Figure 2.**
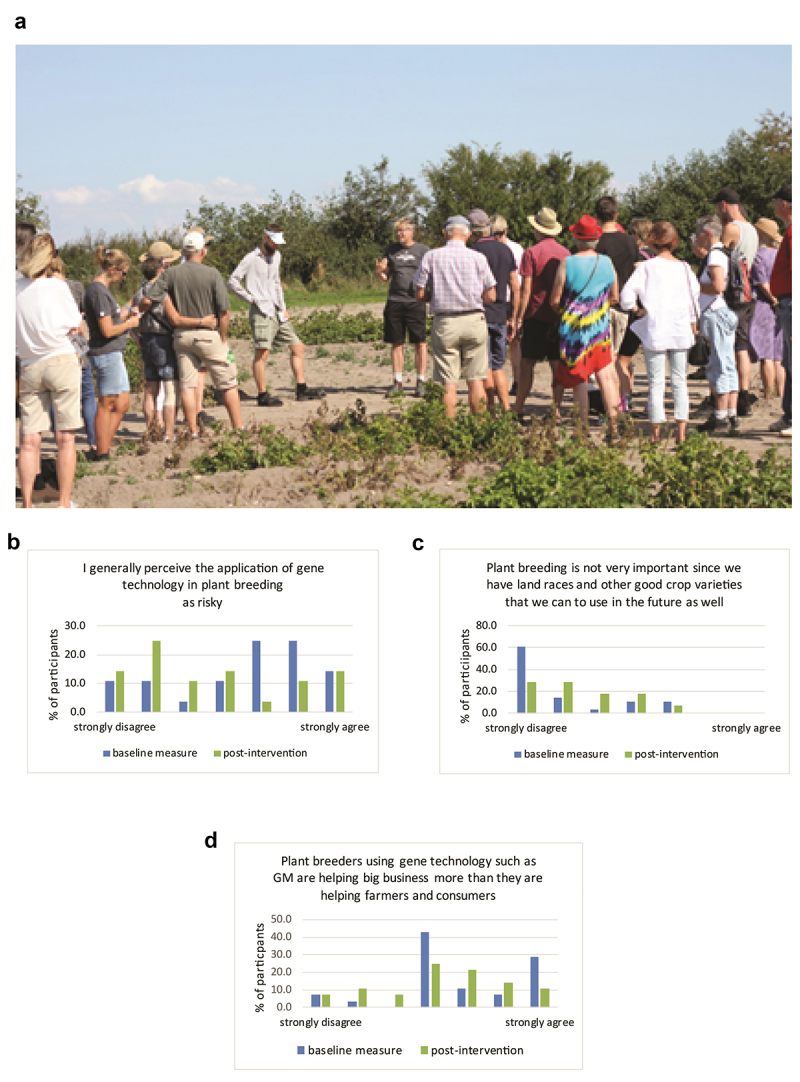
Results of consumers visiting the 3 R-potato field trial on their attitudes. A. Photo of participants and researchers visiting the 3 R-potato field trial. B-D. Descriptive statistics of attitudes, baseline and post-intervention, toward use of gene technology in plant breeding, specifically regarding perception of risk (b), importance for the future (c), and perception of GM foods’ relative helpfulness for big business versus farmers and consumers (d). Results of Repeated Measure ANOVA of differences between baseline and post-intervention scores: F(1,25) = 10.79, p = 0.003; F(1,27) = 4.61, p = 0.041; and F(1,26) = 4.09 p = 0.054, for Figures B, C and D, respectively.

Descriptive statistics of baseline and postintervention measures are presented in Table 1. We found that before the field intervention 65% of the participating consumers perceived GM crops as risky (M = 4.61; SD = 1.93); however, a positive change was observed in the post-intervention assessment (M = 3.62; SD = 2.12) Student’s T-test for paired sample repeated measure statistics confirmed significant mean differences between baseline and post-intervention measure of risk (*I generally perceive the application of gene technology in plant breeding as risky*: t(25) = −3.29, p < 0.01). Within-subjects repeated measure Analysis of Variance (ANOVA) confirmed significant change in risk perception (Fig. 2). Additionally, Student’s T-test for paired sample repeated measure statistics revealed significant mean differences between baseline and post-intervention measures of attitudinal items (*Plant breeding is not very important since we have land races and other good crop varieties that we can use in the future as well* (t(27) = 2.15, p < 0.05) and *Plant breeders using gene technology such as GM are helping big business more than they are helping farmers and consumers* (t(26) = −2.02, p = 0.05)). Within-subjects repeated measure Analysis of Variance (ANOVA) results were significant for these items as well (Fig. 2c-d)

In addition, our results indicate that personal experience and access to a reliable sources of information science in combination with engaged discussion may change consumers’ attitudes for more favorable, shift perception to less risky and therefore increase willingness to accept GM food products. The study, which did not involve any industrial partners, shows that personal experience and a short field intervention can improve perceptions of GM foods and reduce associated stigma. The presented intervention, like any study, is not without limitations. First, the study population was limited and surly not representative to Swedish population, therefore findings need to be interpreted with cautions. However, despite the limited number of participants, we have shown the potential value of an intervention that could be scaled up and used for other types of products. Second, like most other studies regarding GM foods in Europe, this study presents results of hypothetical choices and attitudes toward GM, which might differ from those applied in real purchases.^51^ Nevertheless, despite these limitations it provides clear indications of possible shifts in perceptions associated with field experiences that might be as close as possible to real-life consumers’ experiences with GM products under current laws.

The most widely used method to control late blight is application of fungicides, but extensive use of chemical controls is costly and can be environmentally harmful. Use of the 3 R King Edward lines tested in the study reported here would allow dramatic reductions in costs, since estimated annual costs of fungicides used to control potato late blight in Sweden exceed €440 ha^−1^.^38^ By replacing currently cultivated King Edward cultivars with 3 R cultivars, total use of fungicides in Swedish agriculture could be reduced by several percent, while maintaining the same desired culinary qualities and cultivation routines. Considering that the European Union (EU) recommends to reduce total pesticide use in EU by 50%, our results, clearly show that replacing fungicides with genetic resistance to control late blight could assist to meet this objective.
